# **More Evidence That the Healthcare Administrative Burden Is Real, Widespread and Has Serious Consequences** Comment on "Perceived Burden Due to Registrations for Quality Monitoring and Improvement in Hospitals: A Mixed Methods Study"

**DOI:** 10.34172/ijhpm.2021.129

**Published:** 2021-09-08

**Authors:** Albert J. Heuer

**Affiliations:** Department of Interdisciplinary Studies, Rutgers University, Newark, NJ, USA.

**Keywords:** Administrative Burden, National Quality Forum, Registrations

## Abstract

Countries around the world have implemented programs to help monitor and enhance the quality of health services provided. Inherent in these programs and internal process improvement initiatives are an array of reporting requirements which often place a burden on clinicians and the organizations in which they function. Zegers and colleagues performed a mixed methods study on the perceived burden which these reporting requirements place on doctors, nurses, and other clinicians within three hospitals in the Netherlands. Like all studies, theirs has some minor limitations; most notably possible limits on generalizability from a limited sample. Nonetheless, their project makes a valuable contribution to the growing body of research which suggests that the burden has deleterious effects on clinicians and may well have an erosive impact on patient care.

 The manuscript entitled “*Perceived Burden Due to Registrations for Quality Monitoring and Improvement in Hospitals: A Mixed Methods Study,” *by Zegers et al^1^ focuses is on an important topic related to what they call *registrations,* and what is increasing being referred to in the literature as the *administrative burden* in healthcare. Administrative burden specifically refers to documentation and administrative reporting duties imposed on clinicians due to organizational policies as well as governmental and oversight reporting requirements.^2^ These non-patient-care activities have increased over the past few decades because of many factors which include a variety of quality initiatives stemming from well publicized quality issues and increased emphasis on value-based quality metrics.

 The shift to value-based healthcare, or that which seeks to optimize outcomes which matter to patients in relation to associated costs, is at least partially attributable to the acceptance of the work of experts like Micheal Porter of Harvard Business School.^3^ Inherent in value-based healthcare is data collection and reporting. Hence, some of the administrative burden is warranted and necessary. However, it appears that within the value-based healthcare scheme, more attention may be paid to outcomes than the concomitant reporting requirements, which have proliferated. Because these administrative duties impinge on both the time clinicians have available for direct patient care and organizational resources, such activities may have a deleterious impact on patients and clinical outcomes, and thus may actually undermine value-based healthcare. Furthermore, a study done in the Denmark supports the notion that reporting systems which have a high administrative (compliance) burden, negatively impact work performance and output in healthcare settings. Further, this study also suggests that those systems which are less intuitive and functional tend to undermine morale.^4^ Hence, excessive non-direct patient care duties are increasingly the focus of researchers and scholars, and as a result likely to receive more attention by hospital administrators. Indeed, Zegers et al reinforce important themes published by me and my colleagues in 2016, as well as other research teams, including Lorkowski et al and Rao el al, that the administrative burden is real, widespread, and potentially undermines direct patient care.^2,5,6^

 The compilation of Zegers and similar work points to several of the general drawbacks of excessive, redundant and nonintuitive documentation and reporting requirements. Specifically, they describe the negative impact that registrations categorized by study participants as “unnecessary” and even more harshly rated by others as “unreasonable,” may have on direct-patient care and intrinsic motivation. Furthermore, they cite the work of Shaw et al, Fung et al, Saver et al and others who suggest that some quality reporting programs appear vulnerable to validity and reliability concerns and there is little evidence that such reporting systems and related incentives lead to improved outcomes.^7-9^ Beyond this and consistent with earlier work in this domain, Zegers et al allude to the idea that some of the programs designed to enhance healthcare quality and outcomes may counteract or even detract from such efforts by diluting resources to meet overly onerous reporting requirements. This is especially notable given the immense effort which healthcare organizations expend reporting such data and meeting success thresholds in healthcare systems throughout the developed world.

 Somewhat uniquely, however, Zegers and colleagues’ work distinguishes itself from related research on the overall administrative burden in healthcare, by primarily focusing on a subset of it known as “registration reporting;” or documentation and reporting specifically related to external, third-party oversight organizations and agencies in the Netherlands.^10^ Unlike some other countries including the Unites States, the reporting of quality data is less centralized in the Netherlands.^11^Rather than emanating mainly from a department largely dedicated to obtaining, consolidating and reporting such data on behalf of the organization, the Netherlands uses a less centralized system which also relies on individual clinicians to enter quality data. This places more of a burden on the clinical staff providing direct patient care. It also helps explain why according to Casalino et al, American physicians appear to spend moderately less time on general administrivia and much less time than their Dutch counterparts’ quality (registration) reporting.^12,13^ Another noteworthy characteristic of Zegers and colleagues’ work is that while their survey results did not find an inverse association between the perceptions of unnecessary documentation/reporting and joy-in-work, some interviews they conducted with participants did. Hence, they offer several possible explanations for this conflicting data. Among them that the least palatable rating category for registrations of “unreasonable” was less commonly reported than “unnecessary.” This suggests that a limited amount of the superfluous or redundant registrations and rated as “unnecessary” may be expected and better tolerated than that most harshly rated as “unreasonable.” They also indicate that many participants may be able to rationalize registrations as an undesirable facet of an otherwise rewarding profession.

 The Zegers manuscript is generally well done from a scientific perspective. The topic is potentially of great value to key stakeholders including individual clinicians, healthcare organizations and systems, as well as the patients they serve, many of whom are probably unaware of this phenomenon and the potential threat it poses to the quality of care. The *Methods* seem sound including the study design which facilitates the capture of both quantitative (eg, survey results) data and qualitive (interview) feedback. This mixed methods approach lends itself to more granular feedback to complement the quantitative survey results, aiding in uncovering otherwise hidden themes and more fully exploring the practical significance of the project. As described, the *Results* are a clear, concise, comprehensive and potentially actionable interpretation of both the quantitative and qualitative data ascertained by Zegers et al. The statistical methods appear generally appropriate as they facilitate the exploration of bivariant and multivariant relationships. The *Discussion* and implications stated within it reinforce some of the findings of prior research, including that the phenomenon is real, it might undermine clinician autonomy and morale, which may have downstream impact by eroding the quality of care patient. The implications and recommendations they make are also sound and are themes emanating from healthcare systems of other countries experiencing the same phenomenon. Zegers and colleagues’ recommendations are practical and if implemented would likely result in a better reporting system. Their recommendations include: truncating quality reporting to minimize redundancy (similar to what the National Quality Forum is attempting to help orchestrate in the United States)^14^; involving clinicians and the patients they serve in determining a core set of indicators; sharing the quality data throughout all levels of the organization to provide meaningful context; and altering the culture from *blame and shame* to a more collegial one emphasizing learning and reflection.

 However, like most research, there are a few limitations of this study beyond those described by Zegers et al, which are worthy of mention. Most notably, the project was conducted at only three hospitals, which may not be representative of other hospitals in the Netherlands nor reflective of hospitals or healthcare systems of other countries.^10^ In addition, there was no attempt to compare the structural attributes of these three hospitals and the study participants within them, to those of larger populations. Hence, the ability to generalize these results to other populations, may be limited. In addition, the authors do refer to the incorporation of the “Bern Illegitimate Tasks Scale” and six items from the “Multidimensional Work Motivation Scale” into their ensemble of survey questions, as well as using Cronbach’s alpha to measure reliability. However, there was no description of the resulting Cronbach’s alpha coefficient, only that it was deemed “acceptable.” Further and perhaps more significantly, the means by which the entire survey instrument was validated in the context of the key aims and targeted outcomes of the project was not described. If, in fact, the survey was validated, it would have been best for them to have described the means by which this was accomplished, and the types of validity demonstrated. Lastly, another potential limitation of this project involves the authors’ apparent contradiction regarding whether this project is the first of this type. In the “Strengths and Limitations” section of their manuscript, the authors indicate that to the best of their knowledge this is the first empirical piece on the burden of documentation and reporting specific to quality monitoring and improvement. However, they cite others throughout their manuscript including Botje et al and Andersen et al and indicate under the “Comparison with Earlier Studies” section that “…the studies on registration of quality information are scarce,” suggesting the existence of similar work published by others.^15,16^

 In balance, Zegers et al make an important contribution to the growing body of research in this area and does so in a clear, concise and relevant manner. Many hospitals and healthcare systems around the world face similar challenges of administrative burden in general, and that related to the author’s focus on the documentation and reporting of quality information by clinicians to third-party quality oversight organizations. Indeed, it seems that some of the efforts aimed at enhancing healthcare quality, may have gravitated beyond a cross-over point where the costs now exceed the benefits derived and the now overly burdensome reporting, may have an erosive impact on quality. Hence, the extent to which Zegers et al focuse on a specific subset of the administrative burden and endorses findings of prior research, helps magnify attention paid to the magnitude and implications of the problem, as well as potential remedies. Their recommendations provide stakeholders with potential strategies which can help mitigate this challenge by enhancing organizational efficiency and clinician morale, and quite possibly facilitating better patient care and outcomes. Just as Zegers et al indicate that elements of their project were iterative, so should they and other researchers view their work in this area by building on this project, addressing the few limitations and continuing work in the context of more expansive studies using a validated survey instrument.

## Ethical issues

 The Rutgers University Institutional Review Board (IRB) has approved this project under the “exempt” category.

## Competing interests

 Author declares that he has no competing interests.

## Author’s contribution

 AJH is the single author of the paper.
